# Risk Perception, Acceptance, and Trust of Using AI in Gastroenterology Practice in the Asia-Pacific Region: Web-Based Survey Study

**DOI:** 10.2196/50525

**Published:** 2024-03-07

**Authors:** Wilson WB Goh, Kendrick YA Chia, Max FK Cheung, Kalya M Kee, May O Lwin, Peter J Schulz, Minhu Chen, Kaichun Wu, Simon SM Ng, Rashid Lui, Tiing Leong Ang, Khay Guan Yeoh, Han-mo Chiu, Deng-chyang Wu, Joseph JY Sung

**Affiliations:** 1 Lee Kong Chian School of Medicine Nanyang Technological University Singapore Singapore Singapore; 2 School of Biological Sciences Nanyang Technological University Singapore Singapore; 3 Center for Biomedical Informatics Nanyang Technological University Singapore Singapore; 4 Wee Kim Wee School of Communication and Information Nanyang Technological University Singapore Singapore; 5 The First Affiliated Hospital Sun Yat-sen University Guangzhou China; 6 Xijing Hospital Fourth Military Medical University Xi'an China; 7 Department of Surgery Faculty of Medicine The Chinese University of Hong Kong Hong Kong China (Hong Kong); 8 Prince of Wales Hospital Hospital Authority Hong Kong China (Hong Kong); 9 Department of Gastroenterology and Hepatology Changi General Hospital SingHealth Singapore Singapore; 10 Department of Gastroenterology and Hepatology National University Hospital National University Health System Singapore Singapore; 11 Department of Medicine Yong Loo Lin School of Medicine National University of Singapore Singapore Singapore; 12 Department of Internal Medicine National Taiwan University Hospital Taiwan China; 13 Department of Internal Medicine College of Medicine National Taiwan University Taiwan China; 14 Kaohsiung Medical University Taiwan China

**Keywords:** artificial intelligence, delivery of health care, gastroenterology, acceptance, trust, adoption, survey, surveys, questionnaire, questionnaires, detect, detection, colonoscopy, gastroenterologist, gastroenterologists, internal medicine, polyp, polyps, surgeon, surgeons, surgery, surgical, colorectal

## Abstract

**Background:**

The use of artificial intelligence (AI) can revolutionize health care, but this raises risk concerns. It is therefore crucial to understand how clinicians trust and accept AI technology. Gastroenterology, by its nature of being an image-based and intervention-heavy specialty, is an area where AI-assisted diagnosis and management can be applied extensively.

**Objective:**

This study aimed to study how gastroenterologists or gastrointestinal surgeons accept and trust the use of AI in computer-aided detection (CADe), computer-aided characterization (CADx), and computer-aided intervention (CADi) of colorectal polyps in colonoscopy.

**Methods:**

We conducted a web-based questionnaire from November 2022 to January 2023, involving 5 countries or areas in the Asia-Pacific region. The questionnaire included variables such as background and demography of users; intention to use AI, perceived risk; acceptance; and trust in AI-assisted detection, characterization, and intervention. We presented participants with 3 AI scenarios related to colonoscopy and the management of colorectal polyps. These scenarios reflect existing AI applications in colonoscopy, namely the detection of polyps (CADe), characterization of polyps (CADx), and AI-assisted polypectomy (CADi).

**Results:**

In total, 165 gastroenterologists and gastrointestinal surgeons responded to a web-based survey using the structured questionnaire designed by experts in medical communications. Participants had a mean age of 44 (SD 9.65) years, were mostly male (n=116, 70.3%), and mostly worked in publicly funded hospitals (n=110, 66.67%). Participants reported relatively high exposure to AI, with 111 (67.27%) reporting having used AI for clinical diagnosis or treatment of digestive diseases. Gastroenterologists are highly interested to use AI in diagnosis but show different levels of reservations in risk prediction and acceptance of AI. Most participants (n=112, 72.72%) also expressed interest to use AI in their future practice. CADe was accepted by 83.03% (n=137) of respondents, CADx was accepted by 78.79% (n=130), and CADi was accepted by 72.12% (n=119). CADe and CADx were trusted by 85.45% (n=141) of respondents and CADi was trusted by 72.12% (n=119). There were no application-specific differences in risk perceptions, but more experienced clinicians gave lesser risk ratings.

**Conclusions:**

Gastroenterologists reported overall high acceptance and trust levels of using AI-assisted colonoscopy in the management of colorectal polyps. However, this level of trust depends on the application scenario. Moreover, the relationship among risk perception, acceptance, and trust in using AI in gastroenterology practice is not straightforward.

## Introduction

Artificial intelligence (AI) has made groundbreaking technological advancements in medical image interpretation [1]; diagnosis assistance; risk assessment for various conditions [2]; outcome prognostication [3]; and in certain areas, treatment suggestion [4] and partaking in surgical intervention [5].

Studies of AI trust and acceptance among clinicians are becoming increasingly important. This is because trust and acceptance of AI technology are seen as preconditions for clinical workflow integration [6]. Currently, trust has already been demonstrated by several studies as one of the main determinants in driving the adoption of AI in health care [7,8]. One study showed that within a general home-based health care setting—where AI is applied on the internet of things–based devices to monitor patients’ health—risk perception, acceptance, and trust are related concepts that govern the ultimate use of the developed technology [9]. A separate study [10] conducted on the use of an AI-based system in the application of a Blood Utilization Calculator showed that its trust and use were determined by perceived risk and expectancy (in our context, acceptance). It was demonstrated that high perceived risk reduced trust and subsequent use.

While the clinical evidence of accuracy in the diagnosis and prognosis of AI is accumulating, the level of trust and acceptance by clinicians requires more attention [6]. We identified that gastroenterology, by its very nature of having heavy usage of image-based diagnosis (eg, computed tomography, magnetic resonance imaging, endoscopy, and histology) and surgical or endoscopic intervention, will be one of the specialties that may readily use AI technologies in clinical management [11,12]. Yet, there is little research on AI risk perception, acceptance, and trust among gastroenterologists.

To our knowledge, most published research surveys trust in a more general manner. One such recent example is the survey on gastrointestinal (GI) health care in 2022, which covered clinicians’ perspectives in a general way [13]. However, such surveys lack granularity. It is impossible to know under what circumstances do clinicians become less trusting or accepting or become more concerned about the deployments of AI.

Moreover, there is a lack of explicit modeling from collected data to relate patterns of risk perception, acceptance, and trust among practitioners. There are existing models [14,15] that explore parts of the interactions among these 3 factors. However, because these explorations cover only partial relationships and interactions, we feel that these may be inadequate for modeling real-world dynamics. Therefore, having more comprehensive models would allow for a better understanding of the various factors underpinning how clinicians come to trust, accept, and eventually use AI. This knowledge would help in formulating successful implementation of AI tools in real-world environments.

In this study, we aim to understand the trust and acceptance among gastroenterologists, with a specific focus on the Asia-3Pacific region. We hypothesize is that risk perception, acceptance, and trust will change according to the scenario (computer-aided detection [CADe], computer-aided characterization [CADx], or computer-aided intervention [CADi]), with different levels of invasiveness. A blueprint of a survey that examines contextual responses toward screening colonoscopy with polypectomy in clinical environments is provided. Using our collected data, we attempt to elucidate how risk perception, acceptance, and trust interactions can be modeled and studied. These contributions collectively enhance our understanding of complex factors influencing the integration of AI in medical practice.

## Methods

### Survey

We used a structured questionnaire (Multimedia Appendix 1) to conduct a survey in English by inviting gastroenterologists or GI surgeons from the Asia-Pacific region through open invitations to various medical associations. The questionnaire was based on the expectancy-value framework, major constructs of the Theory of Planned Behaviour research framework [16], and the Technology Acceptance Model measures [17]. Items in the questionnaire for testing risk perception, acceptance, and trust were adapted from various other studies [18,19], with some including items from validated constructs in questionnaires. These questions are then adapted into scenarios covering detection (CADe), characterization (CADx), or intervention (CADi), with different levels of invasiveness characterization and intervention for colonoscopic detection and polypectomy (see Textbox 1 for items used to evaluate these aspects).

Most items were rated on a 7-point Likert scale, where 7 denotes strong agreement. To assess risk perception, acceptance, and trust, we presented participants with 3 different AI applications related to colonoscopy and the management of colorectal polyps. These scenarios, reflecting existing AI applications in GI, involve the detection of polyps (CADe), characterization of the nature of polyps (CADx), and treatment procedures (CADi), respectively (see Table 1 and Textbox 1). Table 2 displays measurement items.

**Figure 1:**
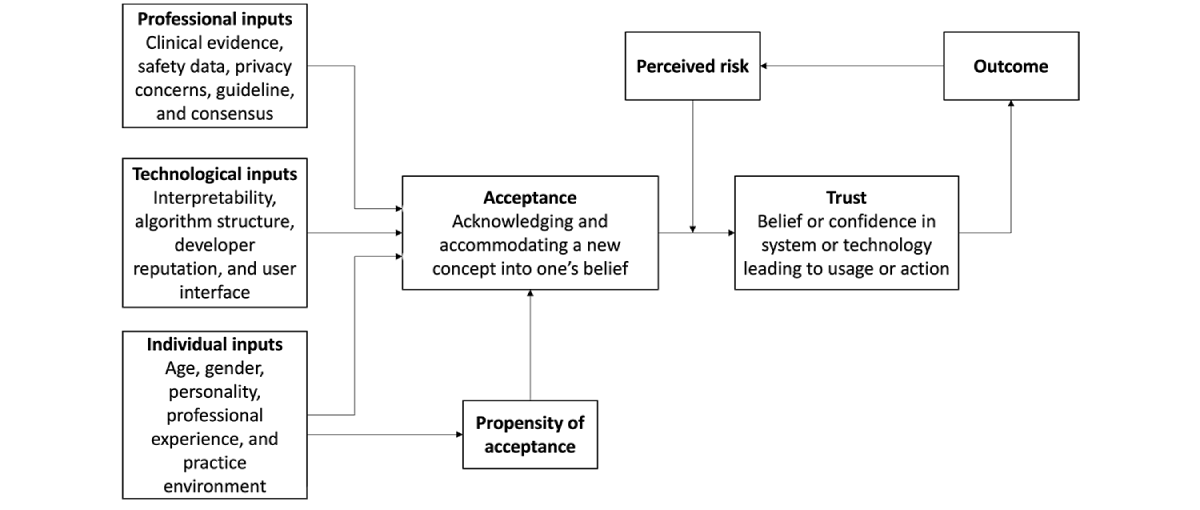
Conceptual model of perceived risk, acceptance, and trust on artificial intelligence decision aids.

In this study, the three key elements for assessment are (1) risk perception, (2) acceptance, and (3) trust. Risk perception refers to an individual’s subjective assessment or understanding of the potential hazards, threats, or uncertainties associated with a particular situation or activity. It involves the process of evaluating and interpreting information about risk, considering factors such as the severity of potential consequences [20,21]. Acceptance is the mental and emotional state of acknowledging and accommodating a new concept or innovation into one’s beliefs, behaviors, or practices. Trust is defined as belief or confidence in the reliability, credibility, and integrity of a person, system, or technology leading to usage or action [20,21]. Acceptance may precede trust in the adoption of new technologies, but trust plays a crucial role in establishing a strong foundation for sustained usage and effective integration of AI into medical practice. Risk perception, acceptance, and trust may interact with each other and other factors stemming from professional, technological, and personal sources. The conceptual framework presented in Figure 1 illustrates the intricate interplay among sociodemographic variables, AI acceptance, trust, perceived risk, and outcomes [22]. Our study aims to contribute to this understanding not by testing individual relationships within this conceptual framework but by exploring how trust, risk, and acceptance are possibly interconnected in the context of AI-supported applications in gastroenterology.

**The 3 operationalized case scenarios of using artificial intelligence–assisted colonoscopy in the management of colorectal polyps.**

**Computer-aided detection**

- Imagine you are attending an informal meeting of colleagues. Your colleagues are not experts in artificial intelligence and have about the same amount of understanding as you do. The conversation turns to innovation in medicine, especially machine learning algorithms and their potential to assist in the interpretation of medical imagery in the early detection of colon cancer. One of the colleagues speaks about a patient who underwent a colonoscopy which was assisted by a machine learning algorithm. When the algorithm indicated that the patient had a colonic polyp, the colleague asked for an additional biopsy. It turned out that the result produced by the algorithm was correct (use the following scale: 1=have major doubts to 4=neutral to 7=fully believe).

**Computer-aided characterization**

- The second colleague reported that the machine learning algorithm is also capable of correctly classifying whether the colonic polyp was adenomatous or hyperplastic (use the following scale: 1=have major doubts to 4=neutral to 7=fully believe).

**Computer-aided intervention**

- Now suppose a third colleague told you that a machine learning algorithm can be applied to guide interventions. Endoscopists need a targeted biopsy from specific locations that harbor the lesion. The third colleague said that the algorithm can guide a biopsy needle more precisely than a human, using ultrasound imaging (use the following scale: 1=have major doubts to 4=neutral to 7=fully believe).

**Table 1:** Scenarios demonstrating AI^a^ use in gastroenterology practice from detection to characterization and intervention.

Scenario	Objective
Computer-aided detection: use of AI to assist in identifying the presence of colorectal polyps and improving adenoma detection rate.	To evaluate the acceptability of AI to assist in the interpretation of medical imagery in detecting colorectal lesions under different bowel preparations and colonic configurations
Computer-aided characterization: use of AI to classify whether a colonic polyp was adenomatous or hyperplastic.	To evaluate the acceptability of AI to differentiate (without histology) between adenoma (with variable degree of malignant potential) vs hyperplastic polyps (no malignant potent)
Computer-aided intervention: use of AI in an endoscopy to guide colonoscopic polypectomy.	To evaluate the acceptability of AI to decide which tool to use in assessing the completeness of polypectomy and risk of bleeding, perforation, or both.

^a^AI: artificial intelligence.

**Table 2:** Survey items used to measure risk perception, acceptance, and trust.

Measure	Question text
Risk perception	I expect major risks involved with the artificial intelligence diagnosis.
Acceptance	Do you believe that machine learning algorithm can, in some cases (as in the one described above), better perform (the task, computer-aided detection, computer-aided characterization, Computer-aided intervention) than human beings?
Trust	I am ready to try the method myself

### Statistical Analysis

Statistically significant application pairs were identified by the Mann-Whitney *U* test (*U* test) or when there is dependence, the Wilcoxon signed-rank test (Wilcox test). Statistical significance is established at .05. Analyses were conducted in Python using the *scipy.stats* module (version 1.10.0; the SciPy community), *statsmodels* module (version 0.13.5), and the *Pingouin* statistical package (version 0.5.3) or SPSS (version 28; IBM Corp).

Correction for multiple testing was performed using Bonferroni correction, where the statistical threshold (α) was divided by the number of tests *n*, such that the adjusted *P* value threshold is given by α/*n*.

### Power Analysis

Our hypothesis is that risk perception, acceptance, and trust will change according to the scenario (detection [CADe], characterization [CADx], or intervention [CADi]), with different levels of invasiveness. Based on an estimated effect size of 0.3 for trust, power, and risk perception with 0.95 power, we can calculate the minimum set of respondents needed to determine any significant differences of a given “size” in response to trust, risk perception, and acceptance measures across scenarios. Since every individual answers scenarios 1 to 3, the differences in the response of every individual can be estimated using a Wilcox test if we compare between pairs of scenarios. The required sample size to pick up a small-moderate effect size (based on Cohen *d*) of 0.3 with a power of 95% is 154. In this study, we have recruited 165 participants, and this should be enough to achieve sufficient statistical power.

### Ethical Considerations

This study was approved by the Nanyang Technological University institutional review board (IRB-2022-756). Informed consent was obtained with ability to opt out. Data was anonymized, and no compensation was provided.

## Results

### Response and Nonresponse Bias

Tracking response rates can help determine the representativeness of a study, but due to the constraints of our institutional review board, we were not allowed to track individual respondents. During the initial phase of the study, we sent the survey to a distribution list of 151 participants with known dates. Applying an approximate 1-month window (October 21, 2022, to November 13, 2022), we obtained 128 responses. Thus, our estimated response rate is ~85% (n=128). While we were analyzing or cleaning up the data, we hoped to get more participants. In the subsequent weeks, we obtained 37 new responses. To compare early and late respondents, we aggregated the first 130 responses (collected between October 21, 2022, and December 29, 2022) as a single group to represent the early respondents and the remaining 35 (collected between January 10 to January 19, 2023) as the late responses. Comparing 130 early respondents against 35 late respondents using a Mann-Whitney *U* test with a Bonferroni-adjusted α=.0056, we found no significant differences for risk, trust, and acceptance across each of the 3 scenarios. This suggests no significant difference between the early and late responses. The lowest obtained *P* value was .022 (trust in CADx), and the remaining *P* values were at least .30. Together, we take these results as a proxy that nonresponder bias is not a strong concern. We also note the overall response rates are rather high; the survey was sent out to various gastroenterology associations as an open invitation, without individual follow-up. It is possible that AI is increasingly seen as transformative and important in the gastroenterology field, but there is not much work on understanding how perspectives on AI lead toward trust and adoption. Hence, invitees feel strongly about the matter and are more inclined to participate in this survey.

### Unidimensionality and Reliability

Most items in our questionnaire were already used in other questionnaires and can be considered as validated. For the scenario-based questions used in this study, these are novel, as we needed to develop new instruments to explore new topics. Participants had to answer on three 7-point items (not at all to wholeheartedly) whether they accept, trust, and perceive risk on the method presented in each of the scenarios. Unidimensionality and reliability were verified and assured using confirmatory factor analysis and Omega Hierarchical, respectively (see Multimedia Appendix 1 for details).

### Cohort Characteristics

In total, 165 clinicians participated in the study. The survey completion rate was ~99.40% (n=165). Participants averaged 44.49 (SD 9.65) years, were mostly male (n=116, 70%), and predominantly specialized in gastroenterology (n=153, 92.72%; see Table 3).

The sample comprised gastroenterologists and GI surgeons with varied clinical experience: 93 (56.36%) participants have over 10 years’ experience in practicing gastroenterology and 111 (66.81%) participants were consultants or senior consultants, mostly working in public hospitals (n=110, 66.67%). Most participants reported basic familiarity with AI (n=160, 96.97%; Q1: How familiar are you with AI?). Many were exposed at work, either directly (n=111, 67.27%; Q2: Have you ever used AI in your occupation?) or indirectly (n=112, 67.88%; Q6: Do you personally know other clinicians who use AI at work?).

Participants rated a mean score of 6.00 (SD 0.95) for intending to use AI when it becomes available in their workplace and a score of 5.50 (SD 1.24) for intending to use it to provide services to their patients. Participants rated a mean score of 5.83 (SD 1.37) for intention to use AI routinely in patient care. These figures suggest generally favorable attitudes toward adopting AI.

**Table 3:** Participant demographics and general characteristics.

Participant		Values (N=165), n (%)
Age (years), mean (SD)^a^		44.49 (9.65)
**Gender^a^**		
	Male	116 (75.32)
	Female	38 (24.68)
**Country or area^b^**		
	Australia	3 (1.83)
	Brunei Darussalam	7 (4.27)
	Hong Kong	18 (10.98)
	India	6 (3.66)
	Indonesia	6 (3.66)
	Japan	9 (5.49)
	New Zealand	1 (0.61)
	People’s Republic of China	50 (30.49)
	Philippines	1 (0.61)
	Republic of Korea	2 (1.22)
	Singapore	24 (14.63)
	Taiwan	33 (20.12)
**Main work setting^c^**		
	Public hospital	110 (67.9)
	Private hospital	28 (17.28)
	Institute of higher learning	18 (11.11)
	Community health center	1 (0.62)
	Other	5 (3.09)
**Current role at work^d^**		
	Resident	19 (11.8)
	Fellow	19 (11.8)
	Consultant	57 (35.4)
	Senior consultant	54 (33.54)
	Other	12 (7.45)
**Specialty^c^**		
	Gastroenterology	153 (94.44)
	Colorectal surgery	4 (2.47)
	General surgery	2 (1.23)
	Other	3 (1.85)
**Practicing in specialty (years)^c^**		
	Less than 5	39 (24.07)
	5-10	30 (18.52)
	11-20	48 (29.63)
	Over 20	45 (27.78)

^a^11 participants did not report their ages or gender.

^b^1 participant did not report their country or area.

^c^3 participants did not report their main work setting, specialty, and years practicing in a specialty.

^d^4 participants did not report their current role at work.

### Scenario-Based Differentiation

**Figure 2:**
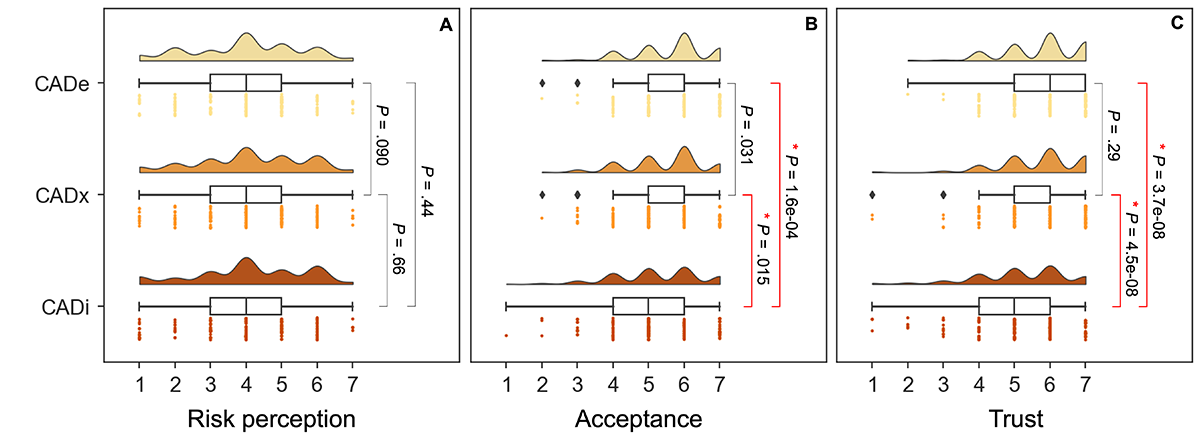
Gastroenterologists’ attitude toward using AI in the management of colorectal polyps: perceived risk, acceptance, and trust in 3 case scenarios of using AI-assisted colonoscopy in CADe, CADx, and adopting CADi with either surgery or endoscopy. Pairwise tests based on the Wilcox test were performed across scenarios. (A) Risk perception across CADe, CADx, and CADi applications. The raincloud plot comprises a 3-panel visualization with a density plot on top revealing density patterns, a box plot in the middle summarizing the median and IQR, and a univariate strip plot on the bottom showing the actual data distribution. No significant pairs were identified. (B) Acceptance across CADe, CADx, and CADi applications. Pairs with statistically significant differences are highlighted by a red connector and an asterisk. (C) Trust across CADe, CADx, and CADi applications. Pairs with statistically significant differences with a *P* value ≤.02 are highlighted by a red connector and an asterisk. AI: artificial intelligence; CADe: computer-aided detection; CADi: computer-aided intervention; CADx: computer-aided characterization.

When participants were exposed to three scenarios in medical practice that extend from (1) diagnosing and detecting colorectal polyps (CADe), (2) assessing the nature of pathology of polyps and predict risk of malignancy (CADx), and (3) adopting endoscopic or surgical intervention or removal of the polyps (CADi), clinicians expressed similar risk perceptions across all applications (Figure 2A: Median_CADe_=Median_CADx_=Median_CADi_=4.0; Wilcox_CADe-CADx_: *P*=.09; Wilcox_CADe-CADi_: *P*=.44; Wilcox_CADx-CADi_: *P*=.66). However, there were clear application-specific differences in intention to accept AI in practice, with CADe and CADx rated higher than that of CADi (Figure 2B: Median_CADe_=6.0, Median_CADx_=6.0, Median_CADi_=5.0; Wilcox_CADe-CADx_: *P*=.031; Wilcox_CADe-CADi_: *P*=1.6×10^–4^; Wilcox_CADx-CADi_: *P*=.02). Similarly for trust, CADe and CADx were rated higher than CADi (Figure 2C: Median_CADe_=6.0, Median_CADx_=6.0, Median_CADi_=5.0; Wilcox_CADe-CADx_: *P*=.29; Wilcox_CADe-CADi_: *P*=3.7×10^–08^; Wilcox_CADx-CADi_: *P*=4.5×10^–08^).

### Subgroup Analysis for Identification of Confounding Effects and Other Intrinsic Factors

**Figure 3:**
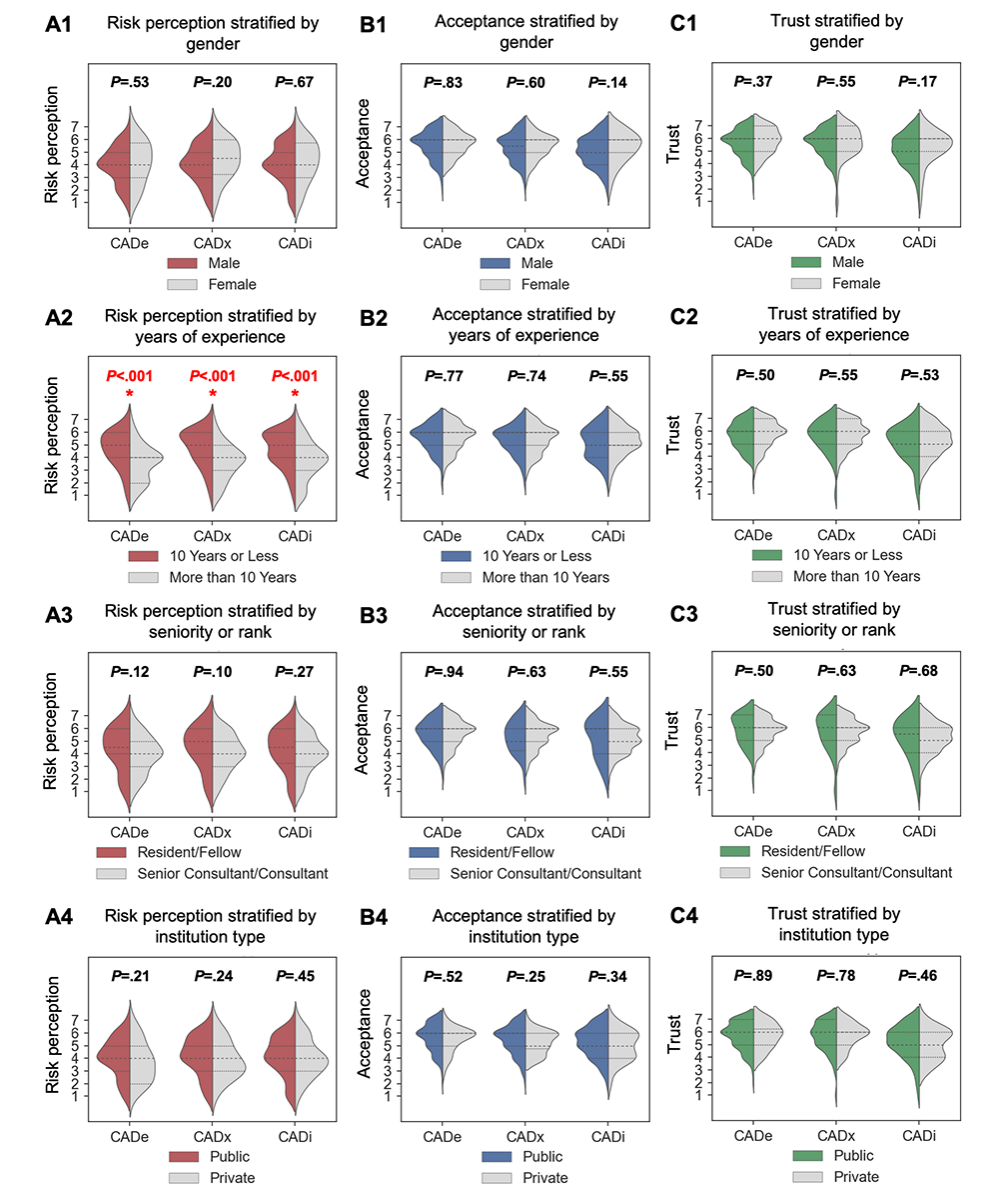
Subgroup analysis of risk perception, acceptance, and trust stratified by year of experience, seniority (consultant+senior consultant vs fellow+resident), gender, and practicing environment (public vs private hospital). The visualization is a grouped violin plot with split violins. The left and right halves of the violin depict the distributions of 2 samples. If the 2 samples are similar, they will exhibit symmetry on both sides. The median lines for each sample have dashed lines, and these median lines are in turn, bordered by their respective 25th and 75th percentile lines depicted as dotted horizontal lines. Comparisons with statistically significant differences with *P* value ≤.0014 are flagged with a red asterisk. CADe: computer-aided detection; CADi: computer-aided intervention; CADx: computer-aided characterization.

We performed a subgroup analysis to investigate if factors such as gender, years of experience, and practice environment will affect risk perception, acceptance, and trust in AI for gastroenterology practice (Figure 3).

Male and female practitioners held similar risk perceptions. There was good concordance in their risk perception, acceptance, and trust toward using AI in gastroenterology practice (Figure 3A1, 3B1, and 3C1). Male participants tended to be less accepting and trusting, especially in CADi, although this difference is not statistically significant.

Next, we compared practitioners with 10 or less years of clinical experience (n=69) versus experienced practitioners with more than 10 years of clinical experience (n=93). While the overall trends of high acceptance and trust showed no difference between the 2 groups, experienced clinicians exhibited consistently lower risk perception than less experienced ones (Figure 3A2). This observation was statistically significant for all 3 scenarios (CADe: *P*=9.7×10^–6^; CADx: *P*=1.7×10^–06^; CADi: *P*=3.3×10^–04^). We also compared practitioners of the rank senior consultant and consultant (n=111) against residents and fellows (n=38; Figure 3A3, 3B3, and 3C3). The acceptance and trust remained high, and the trend showed a good concordance between the 2 groups. A lower risk perception was found among senior consultants and consultants compared to residents and fellows (CADe: *P*=.12, CADx: *P*=.10, and CADi: *P*=.27). However, the difference is statistically insignificant. The years of experience in clinical practice appeared to have a stronger impact on risk perception than the rank held.

Finally, we compared practitioners from public hospitals with those from private hospitals (Figure 3A4, 3B4, and 3C4). There was no statistically significant difference between private hospital practitioners against their public counterparts, although there was a noticeable difference in CADx on acceptance (Figure 3B4). There was also a lower rate of acceptance and trust in using AI for intervention (CADi) compared to CADe and CADx. Despite not reaching statistical significance, we observed that the spread among private hospital respondents tended to exhibit greater variations. In some instances, the spread appeared to be bimodal for CADi, suggesting that the private respondents could be a combination of 2 distinct subgroups.

The correlation among risk perception, acceptance, and trust was further analyzed by incorporating the years of experience of the participants by their years of practice in gastroenterology. In all 3 scenarios, there is a moderate correlation between acceptance and trust of AI in detecting polyps (CADe) and characterizing polyps (CADx). The influence of risk perception on acceptance and trust appears to be more diffused: noticeably, when trust and acceptance are both high, and it does not always coincide with low-risk perception.

We first used contingency tables combined with the Fisher exact test to evaluate the impact on the original relationships between trust and acceptance and after introducing risk perception (risk) as an interaction term. This was repeated for each scenario (CADx, CADi, and CADe; Multimedia Appendix 1). Using this approach, we find that after introducing risk perception, the distribution of values still largely follows that of the original data, suggesting that risk does not interact strongly with trust and acceptance. However, this does not mean that risk does not influence these 2 factors. To further investigate, we performed a 2-way ANOVA to further study the influence of risk perception on acceptance and trust. The 2-way ANOVA revealed a statistically significant interaction in CADe (*F*_25_=3.37; *P*=1.6×10^–05^) but not in CADx (*F*_25_=1.40; *P*=.12) and CADi (*F*_36_=1.35; *P*=.16). Finally, we performed two sets of regression analyses with (1) acceptance and risk perception as independent variables and (2) acceptance, risk perception, and an interaction term that is the product of acceptance and risk perception (Multimedia Appendix 1). Acceptance had a statistically significant positive influence on trust for all 3 scenarios. Risk perception only has a statistically significant negative impact on trust for the first 2 scenarios (CADe and CADx). When we considered an interaction term, only CADe had a statistically significant impact on trust on all 3 terms. For CADx and CADi, this effect disappeared and only acceptance retained a statistically significant influence on trust. Thus, we believe risk perception has a weak association with trust and acceptance. Taken together, the relationship between trust, acceptance, and risk perception appears complex and is not straightforward.

## Discussion

### Principal Findings

The findings from our study demonstrate that gastroenterologists are generally familiar with AI and were frequently exposed to AI tools in medical settings. This may be because of the introduction of AI-assisted colonoscopy by various industries. In recent years, there are also numerous publications and seminars in the field of gastroenterology mentioning the success of using AI tools in diagnosis, risk prediction, and the treatment of GI conditions [23]. This suggests that they have a keen awareness of AI’s future potential in clinical applications. However, our findings showed that acceptance is not an all-or-nothing choice, but the application or intention to use AI tools varied between different clinical scenarios as well as the nature and impact of AI participation.

When looking at scenario-specific acceptance and trust in AI, the responses vary. Our survey on AI use in detection (CADe), characterization (CADx), and intervention (CADi) of colonic polyps revealed wide acceptance disparity among practitioners (Figure 2). While CADe was more widely accepted, CADi was met with much greater resistance. The 3 AI scenarios that were presented to clinicians in this study varied in the degree of involvement a clinician has in certain procedures. Participants preferred CADi the least. These results agree with our hypothesis that trust, acceptance, and risk perception will change according to the scenario (detection [CADe], characterization [CADx], or intervention [CADi]), with different levels of invasiveness.

In this study, acceptance appeared to have little correlation with the perceived risk level of the procedures. Although certain case scenarios were considered by some as high risk, they do not necessarily warrant low acceptance or trust in using AI. Hence, the findings highlight the intricate relationship between the complexity of AI technologies and their acceptance. One intriguing finding is that participants with more (years of) experience appear to accept the risk and would trust the use of AI more than those who are less experienced. This probably indicates that they see the use of AI as an option or recommendation, instead as an obligation or necessity. Therefore, having more clinical experience may give clinicians greater confidence in their medical expertise and practice, thereby generating more confidence in risk mitigation when new technologies are introduced. Indeed, a study by Lawton et al [24] revealed more experienced doctors were much more at ease with uncertainty.

On the other hand, a general lack of AI familiarization and training in medical education may be one of the reasons that less experienced doctors perceive AI as more risky than regular or traditional practice. Chen et al [25] found that while most physicians and medical students were receptive to the use of AI, most also had concerns about the potential for unpredictable or incorrect results. The same study also stated that respondents were aware of AI’s potential but lacked practical experience and related knowledge. Thus, introducing AI literacy and familiarization training early in medical careers may help mitigate risk aversion and promote responsible AI use in clinical practice. Young doctors are also aware of their education gaps. In a study by Civaner et al [26], medical student respondents acknowledged a gap in “knowledge and skills related to AI applications” (96.2%), “applications for reducing medical errors” (95.8%), and “training to prevent and solve ethical problems that might arise as a result of using AI applications” (93.8%).

Our results suggest that although there is a moderate correlation between trust and acceptance, risk perception appeared invariant suggesting the relationship between trust and acceptance with risk perception is not straightforward and may implicate other factors and interactions than the relationships shown in Figure 1. Indeed, the invariance of risk perception across scenarios against acceptance suggests that there are other factors that influence the acceptance of AI (Figure 2). Among the tested factors, we find that risk acceptance is confounded with years of experience (Figure 3). Future studies should be conducted to better understand other drivers and barriers that influence acceptance, such as the perceived usefulness of using AI and whether AI tools may replace the jobs of clinicians in future practices. Qualitative studies, such as the use of focus group discussions, would also be useful to better understand clinicians’ specific concerns in using AI and the impact of their concerns on the use of AI. Quantitatively, more complex data analysis methods may also be used in the future to understand the causal relationship between various factors and the acceptance of AI. As we proceed into deeper and larger cohort studies investigating trust and acceptance of AI, the development of powerful network methodologies can yield more insight. Indeed, simple statistical learning and even deep learning methods may soon become limited in their ability to explain complex and directed relationships among factors. We believe that causal analysis methods, such as Bayesian Belief Networks will soon become necessary and indispensable for explaining and modeling trust, acceptance, and risk perceptions on medical AI [27].

### Limitations

There are limitations in this study. While this study provides invaluable insight into the Asia-Pacific region, we have only captured clinicians’ perspectives despite there being other stakeholders whose voices and opinions matter. This includes nurses, endoscopy assistants, and patients. Future studies should aim to capture their perspectives and understand better how their opinions align or conflict with each other. This will help us navigate complex trust and acceptance issues more realistically and create valuable propositions and effective policies by adopting a multistakeholder perspective into consideration [28]. Participants in this study come from 5 countries with only 165 respondents. The generalizability of the findings can be strengthened by including more clinicians from different backgrounds and regions of practice. In future implementation studies, it may also be worthwhile to examine additional case scenarios such as the management of complicated inflammatory bowel diseases; choice of therapy for GI cancers and GI bleeding; and their corresponding trust, acceptance, and risk perceptions. This additional information will help us better contextualize how risk acceptance, acceptance, and trust change depending on practice.

### Conclusions

This study is one of the first to examine risk perception, acceptance, and trust across different scenarios. It is one of the earliest reports of AI risk perception, acceptance, and trust among gastroenterologists, with a unique focus on the Asia- Pacific region. We found that gastroenterologists have, in general, a high acceptance and trust level of using AI-assisted colonoscopy in the management of colorectal polyps. However, this level of trust depends on the application scenario. Moreover, the relationship among risk perception, acceptance, and trust in using AI in gastroenterology practice is not a straightforward correlation. Future studies are required to identify factors that influence the acceptance and trust of using AI in clinical practices.

## Appendix

Multimedia Appendix 1 Survey questions and supplementary results.

## Abbreviations

AI: artificial intelligence
CADe: computer-aided detection
CADi: computer-aided intervention
CADx: computer-aided characterization
GI: gastrointestinal
