# Supplementary material for: Risk Perception, Acceptance, and Trust of Using AI in Gastroenterology Practice in the Asia-Pacific Region: Web-Based Survey Study
Source: JMIR AI. 2024 Mar 7;3:e50525. doi: 10.2196/50525 (PMC11041476; doi:10.2196/50525)
Supplement: Multimedia Appendix 1 [file ai_v3i1e50525_app1.docx]

**Supplementary Material: Survey Questions**

| ID | Automatically generated |
| --- | --- |
| Country |  |
| Hospital |  |

Q1. Artificial Intelligence (AI) is a new development in healthcare that has been implemented in various areas including in gastroenterology. It is an umbrella term encompassing various technologies (e.g., “Machine Learning”) and broadly refers to the ability of computers to perform tasks that traditionally require human intelligence such as learning and problem-solving. How familiar are you with AI?

| Not familiar at all | Slightly familiar | Moderately familiar | Very familiar |
| --- | --- | --- | --- |
| 1 | 2 | 3 | 4 |

Q2. Have you ever used AI in your occupation?

- Yes
- No

Q3. What do you expect will happen when AI is increasingly used in gastroenterological diagnosis? Please use the following scale: 1 = Impossible to happen to 7 = Certain to happen

|  | Impossible to happen |  |  |  |  |  | Certain to happen |
| --- | --- | --- | --- | --- | --- | --- | --- |
| 1. The quality of care will increase. | 1 | 2 | 3 | 4 | 5 | 6 | 7 |
| 1. There will be fewer medical errors. | 1 | 2 | 3 | 4 | 5 | 6 | 7 |
| 1. AI will improve diagnostic efficiency over the next years. | 1 | 2 | 3 | 4 | 5 | 6 | 7 |
| 1. AI (combined with human inputs) will decrease errors. | 1 | 2 | 3 | 4 | 5 | 6 | 7 |
| 1. AI will improve the timeliness of patient care (i.e., improving the promptness of patient care without compromising quality). | 1 | 2 | 3 | 4 | 5 | 6 | 7 |
| 1. AI will make care more cost-efficient. | 1 | 2 | 3 | 4 | 5 | 6 | 7 |
| 1. AI will increase the workload of clinicians. | 1 | 2 | 3 | 4 | 5 | 6 | 7 |
| 1. AI will enhance patients’ welfare (e.g., patients’ state of doing well in areas such as happiness, well-being, or prosperity). | 1 | 2 | 3 | 4 | 5 | 6 | 7 |
| 1. AI will eventually replace pathologists. | 1 | 2 | 3 | 4 | 5 | 6 | 7 |
| 1. AI will hinder clinician-patient relationship. | 1 | 2 | 3 | 4 | 5 | 6 | 7 |
| 1. AI will bring psychological harm to the patient. | 1 | 2 | 3 | 4 | 5 | 6 | 7 |
| 1. The methods used for developing AI algorithm will be transparent. | 1 | 2 | 3 | 4 | 5 | 6 | 7 |
| 1. Computer-assisted polyp detection will improve endoscopic performance. | 1 | 2 | 3 | 4 | 5 | 6 | 7 |
| 1. Doctors will lose control over diagnosis and treatment of their patients. | 1 | 2 | 3 | 4 | 5 | 6 | 7 |

Q4. And how much do you agree with the following statements?

Use the following scale: 1 = Completely disagree to 7 = Completely agree

|  | Completely disagree |  |  |  |  |  | Completely agree |
| --- | --- | --- | --- | --- | --- | --- | --- |
| 1. Learning to operate AI technology would NOT be easy for me. | 1 | 2 | 3 | 4 | 5 | 6 | 7 |
| 1. Using AI technology will be clear and understandable for me. | 1 | 2 | 3 | 4 | 5 | 6 | 7 |
| 1. If tested properly, the findings of AI can be trusted. | 1 | 2 | 3 | 4 | 5 | 6 | 7 |
| 1. If licensed properly, the findings of AI can be trusted. | 1 | 2 | 3 | 4 | 5 | 6 | 7 |
| 1. AI-assisted prognostic assessment is helpful. | 1 | 2 | 3 | 4 | 5 | 6 | 7 |
| 1. Regardless of the AI, the final responsibility lies with the clinician. | 1 | 2 | 3 | 4 | 5 | 6 | 7 |
| 1. The developer of the technology bears joint responsibility. | 1 | 2 | 3 | 4 | 5 | 6 | 7 |
| 1. Algorithms will be always open to biases. | 1 | 2 | 3 | 4 | 5 | 6 | 7 |
| 1. Patients should be informed about the use of AI. | 1 | 2 | 3 | 4 | 5 | 6 | 7 |
| 1. Patients should actively agree to the use of AI. | 1 | 2 | 3 | 4 | 5 | 6 | 7 |
| 1. Prognostic information from AI should only be available to clinicians. | 1 | 2 | 3 | 4 | 5 | 6 | 7 |
| 1. Prognostic statements on therapy should only be available to the clinicians. | 1 | 2 | 3 | 4 | 5 | 6 | 7 |

Q5. And how much do you agree with the following statements?

Do use again the following scale: 1 = Completely disagree to 7 = Completely agree

|  | Completely disagree |  |  |  |  |  | Completely agree |
| --- | --- | --- | --- | --- | --- | --- | --- |
| 1. Using AI may decrease my control over clinical decisions. | 1 | 2 | 3 | 4 | 5 | 6 | 7 |
| 1. Using AI may decrease my professional discretion over patient care decisions. | 1 | 2 | 3 | 4 | 5 | 6 | 7 |
| 1. Using AI can decrease my control over each step of the patient care process. | 1 | 2 | 3 | 4 | 5 | 6 | 7 |
| 1. Using AI may increase non-providers (e.g., other hospital staff) monitoring my diagnostic and therapeutic decisions. | 1 | 2 | 3 | 4 | 5 | 6 | 7 |
| 1. Using AI may decrease my control over the allocation of scarce resources. | 1 | 2 | 3 | 4 | 5 | 6 | 7 |

Q6. Do you personally know other clinicians who use AI at work?

- Yes
- No

Q7. To what extent would the clinicians who use AI that you are in close contact with, agree with the following statements?

Use the following scale: 1 = Strongly disagree to 4 = Neutral to 7 = Strongly agree

|  | Strongly disagree |  |  | Neutral |  |  | Strongly agree |
| --- | --- | --- | --- | --- | --- | --- | --- |
| 1. Using AI is easy. | 1 | 2 | 3 | 4 | 5 | 6 | 7 |
| 1. AI is an effective tool. | 1 | 2 | 3 | 4 | 5 | 6 | 7 |

Q8. Who or what will have an impact on your decision to use AI routinely in your practice? For each of the entries below, on a scale of 0-10, how strong do you assess the influence to be?

1. The head of my department in my hospital
2. The company that built and sells the product
3. The outcome of a series of tests run using AI
4. The general opinion among doctors
5. The general opinion among patients
6. How much it costs

Q9. Assuming clear evidence of benefit to patients, what do you feel is most important for healthy adoption of AI in gastroenterology?

Use the following scale: 1 = Not important at all to 7 = Very important

|  | Not important at all |  |  |  |  |  | Very important |
| --- | --- | --- | --- | --- | --- | --- | --- |
| 1. Improvement to efficiency in workflow | 1 | 2 | 3 | 4 | 5 | 6 | 7 |
| 1. Reduction of medical legal risk | 1 | 2 | 3 | 4 | 5 | 6 | 7 |
| 1. Reproducible outcomes | 1 | 2 | 3 | 4 | 5 | 6 | 7 |
| 1. Reduction of physician fatigue | 1 | 2 | 3 | 4 | 5 | 6 | 7 |

In the following, we present you three different scenarios. Please, read them **carefully**. After that we will ask you some questions about these scenarios.

Q10. Scenario 1

Imagine you are attending an informal meeting of colleagues. Your colleagues are not experts in AI and have about the same amount of understanding as you do. The conversation turns to innovation in medicine, especially machine learning algorithms and their potential to assist in the interpretation of medical imagery in the early detection of colon cancer. One of the colleagues speaks about a patient who underwent a colonoscopy which was assisted by a machine learning algorithm. When the algorithm indicated that the patient had a colonic polyp, the colleague asked for an additional biopsy. It turned out that the result produced by the algorithm was correct.

Use the following scale: 1 = Have major doubts to 4 = Neutral to 7 = Fully believe

|  | Have major doubts |  |  | Neutral |  |  | Fully believe |
| --- | --- | --- | --- | --- | --- | --- | --- |
| 1. Do you fully believe the colleague, or will you harbor doubts? | 1 | 2 | 3 | 4 | 5 | 6 | 7 |
| 1. Do you believe that machine learning algorithm can, in some cases (as in the one described above), better identify a polyp than human beings? | 1 | 2 | 3 | 4 | 5 | 6 | 7 |

 Use the following scale: 1 = Not at all to 4 = Neutral to 7 = Wholeheartedly

|  | Not at all |  |  | Neutral |  |  | Wholeheartedly |
| --- | --- | --- | --- | --- | --- | --- | --- |
| 1. Will you accept the method? | 1 | 2 | 3 | 4 | 5 | 6 | 7 |

Use the following scale: 1 = Strongly disagree to 4 = Neutral to 7 = Strongly agree

|  | Strongly disagree |  |  | Neutral |  |  | Strongly agree |
| --- | --- | --- | --- | --- | --- | --- | --- |
| 1. I am ready to try the method myself. | 1 | 2 | 3 | 4 | 5 | 6 | 7 |
| 1. I expect major risks involved with the AI diagnosis. | 1 | 2 | 3 | 4 | 5 | 6 | 7 |

Q11. Scenario 2

The second colleague reported that the machine learning algorithm is also capable of correctly classifying whether the colonic polyp was adenomatous or hyperplastic.

Use the following scale: 1 = Have major doubts to 4 = Neutral to 7 = Fully believe

|  | Have major doubts |  |  | Neutral |  |  | Fully believe |
| --- | --- | --- | --- | --- | --- | --- | --- |
| 1. Do you fully believe the colleague, or will you harbor doubts? | 1 | 2 | 3 | 4 | 5 | 6 | 7 |
| 1. Do you believe that machine learning algorithm can, in some cases (as the one described above better), classify a polyp than human beings? | 1 | 2 | 3 | 4 | 5 | 6 | 7 |

 Use the following scale: 1 = Not at all to 4 = Neutral to 7 = Wholeheartedly

|  | Not at all |  |  | Neutral |  |  | Wholeheartedly |
| --- | --- | --- | --- | --- | --- | --- | --- |
| 1. Will you accept the method? | 1 | 2 | 3 | 4 | 5 | 6 | 7 |

Use the following scale: 1 = Strongly disagree to 4 = Neutral to 7 = Strongly agree

|  | Strongly disagree |  |  | Neutral |  |  | Strongly agree |
| --- | --- | --- | --- | --- | --- | --- | --- |
| 1. I am ready to try the method myself. | 1 | 2 | 3 | 4 | 5 | 6 | 7 |
| 1. I expect major risks involved with the AI diagnosis. | 1 | 2 | 3 | 4 | 5 | 6 | 7 |

Q12. Scenario 3

Now suppose a third colleague told you that a machine learning algorithm can be applied to guide interventions. Endoscopists need a targeted biopsy from specific locations that harbor the lesion. The third colleague said that the algorithm can guide a biopsy needle more precisely than a human, using ultrasound imaging.

Use the following scale: 1 = Have major doubts to 4 = Neutral to 7 = Fully believe

|  | Have major doubts |  |  | Neutral |  |  | Fully believe |
| --- | --- | --- | --- | --- | --- | --- | --- |
| 1. Do you fully believe the colleague, or will you harbor doubts? | 1 | 2 | 3 | 4 | 5 | 6 | 7 |
| 1. Do you believe that machine learning algorithm can, in some cases (as the one described above), guide the needle better than human beings? | 1 | 2 | 3 | 4 | 5 | 6 | 7 |

 Use the following scale: 1 = Not at all to 4 = Neutral to 7 = Wholeheartedly

|  | Not at all |  |  | Neutral |  |  | Wholeheartedly |
| --- | --- | --- | --- | --- | --- | --- | --- |
| 1. Will you accept the method wholeheartedly? | 1 | 2 | 3 | 4 | 5 | 6 | 7 |

Use the following scale: 1 = Strongly disagree to 4 = Neutral to 7 = Strongly agree

|  | Strongly disagree |  |  | Neutral |  |  | Strongly agree |
| --- | --- | --- | --- | --- | --- | --- | --- |
| 1. I am ready to try the method myself. | 1 | 2 | 3 | 4 | 5 | 6 | 7 |
| 1. I expect major risks involved with the AI diagnosis. | 1 | 2 | 3 | 4 | 5 | 6 | 7 |

Q13. How does it feel for you that AI technology will be used in patient care?

|  | 1 | 2 | 3 | 4 | 5 | 6 | 7 |  |
| --- | --- | --- | --- | --- | --- | --- | --- | --- |
| Bad |  |  |  |  |  |  |  | Good |
| Frightening |  |  |  |  |  |  |  | Reassuring |
| Worthless |  |  |  |  |  |  |  | Valuable |
| Unpleasant |  |  |  |  |  |  |  | Pleasant |
| Dull |  |  |  |  |  |  |  | Exciting |
| Harmful |  |  |  |  |  |  |  | Beneficial |

Q14. Thinking about the near future, how would you rate the following statements?

Use the following scale: 1 = Completely disagree to 4 = Neutral to 7 = Completely agree

|  | Strongly disagree |  |  | Neutral |  |  | Strongly agree |
| --- | --- | --- | --- | --- | --- | --- | --- |
| 1. I intend to use AI technology in my patient care when it becomes available in my department or hospital. | 1 | 2 | 3 | 4 | 5 | 6 | 7 |
| 1. Disregarding cost, I intend to use AI technology to provide health-care services to patients as often as needed. | 1 | 2 | 3 | 4 | 5 | 6 | 7 |
| 1. I intend NOT to use AI technology in my patient care routinely. |  |  |  |  |  |  |  |

| Q15. What are other concerns regarding the application of AI in your occupation that we have not yet addressed? |
| --- |
| Response: |

Q16. What is your gender?

- Male
- Female

Q18. What is your age? ______________ (Text box with number input only)

Q19. What is your specialty?

- Gastroenterology
- Colorectal surgery
- General surgery
- Other (please specify): ________________(Open text box)

Q20. How many years (total full-time equivalent) have you have been practicing in your specialty?

- <5 years
- 5-10 years
- 11-20 years
- > 20 years

Q21. What is your current work role at work?

- Resident
- Fellow
- Consultant
- Senior Consultant
- Other (please specify): ________________(Open text box)

Q22. What is your main work setting?

- Public hospital
- Private hospital
- Community health center
- Institutes of higher learning
- Other (please specify): ________________(Open text box)

Q23. How many practicing physicians are the department of your primary appointment?

- < 5 staff
- 5-10 staff
- 11-20 staff
- >20 staff

If you would like to be contacted regarding future studies, please check the box below. I allow the researchers to contact me and inform me about future studies.

- Yes
- No

All the information that you have shared with us very helpful. Thank you for your participation in our study!

Supplementary Results

**Result 1 FISHER’s Exact Test on Contingency Tables**

We used contingency tables combined with the Fisher’s exact test to evaluate impact on the original relationships between Trust and Acceptance, and after introducing Risk Perception (Risk) as an interaction term. We repeated this for each scenario (CADx, CADi and CADe)

**Scenario 1 (CADe)**

| [Overall] | Low Acceptance | High Acceptance |
| --- | --- | --- |
| High Trust | 19 | 122 |
| Low Trust | 9 | 15 |

statistic: 0.26

p-value: 0.0075

| [High Risk] | Low Acceptance | High Acceptance | [Low Risk] | Low Acceptance | High Acceptance |
| --- | --- | --- | --- | --- | --- |
| High Trust | 8 | 44 | High Trust | 11 | 78 |
| Low Trust | 0 | 9 | Low Trust | 9 | 6 |

[High Risk]

statistic: inf

p-value: 0.59

[Low Risk]

statistic: 0.094

p-value: 0.00016

**Scenario 2 (CADx)**

| [Overall] | Low Acceptance | High Acceptance |
| --- | --- | --- |
| High Trust | 19 | 122 |
| Low Trust | 16 | 8 |

statistic: 0.0778688524590164

p-value: 1.54e-07

| [High Risk] | Low Acceptance | High Acceptance | [Low Risk] | Low Acceptance | High Acceptance |
| --- | --- | --- | --- | --- | --- |
| High Trust | 10 | 48 | High Trust | 9 | 74 |
| Low Trust | 3 | 6 | Low Trust | 13 | 2 |

[High Risk]

statistic: 0.42

p-value: 0.36

[Low Risk]

statistic: 0.019

p-value: 7.89e-09

**Scenario 3 (CADi)**

| [Overall] | Low Acceptance | High Acceptance |
| --- | --- | --- |
| High Acceptance | 13 | 106 |
| Low Acceptance | 33 | 13 |

statistic: 0.048

p-value: 4.71e-14

| [High Risk] | Low Acceptance | High Acceptance | [Low Risk] | Low Acceptance | High Acceptance |
| --- | --- | --- | --- | --- | --- |
| High Trust | 3 | 45 | High Trust | 10 | 61 |
| Low Trust | 12 | 5 | Low Trust | 21 | 8 |

[High Risk]

statistic: 0.028

p-value: 5.29e-07

[Low Risk]

statistic: 0.062

p-value: 3.17e-08

**Result 2 Consistency and Uni-dimensionality Checks**

These are results from CFI and estimates of the reliability of a multi-item scale using more modern methods than coefficient alpha, that is an index called omega hierarchical for interval-level item response metrics (cf. Kelley and Pomprasertmani, 2016).

| **Consistency and Unidimensionality Check for Acceptance** | | |  |  |
| --- | --- | --- | --- | --- |
| Internal Consistency: Omega Hierarchical | | |  |  |
| **Sample Size** | **Estimate** | **Std Error** | **95% Lower Limit** | **95% Upper Limit** |
| 165 | 0.758 | 0.037 | 0.680 | 0.827 |
|  |  |  |  |  |
| Internal Consistency: Pearson r | |  |  |  |
| **Lowest r** | **Highest r** | **0.25 quantile r** | **Median r** | **0.75 quantile r** |
| 0.380 | 0.573 | 0.462 | 0.545 | 0.559 |
|  |  |  |  |  |
| Test of Unidimensionality: Confirmatory Factor Analysis | | |  |  |
| **Factor** | **Factor Loadings** |  |  |  |
| CADe Acceptance | 0.628 |  |  |  |
| CADx Acceptance | 0.977 |  |  |  |
| CADi Acceptance | 0.779 |  |  |  |
|  |  |  |  |  |

|  |  |  |  |  |
| --- | --- | --- | --- | --- |
| **Consistency and Unidimensionality Check for Trust** | | |  |  |
| Internal Consistency: Omega Hierarchical | | |  |  |
| **Sample Size** | **Estimate** | **Std Error** | **95% Lower Limit** | **95% Upper Limit** |
| 165 | 0.843 | 0.025 | 0.789 | 0.886 |
|  |  |  |  |  |
| Internal Consistency: Pearson r | |  |  |  |
| **Lowest r** | **Highest r** | **0.25 quantile r** | **Median r** | **0.75 quantile r** |
| 0.488 | 0.789 | 0.559 | 0.631 | 0.710 |
|  |  |  |  |  |
| Test of Unidimensionality: Confirmatory Factor Analysis | | |  |  |
| **Factor** | **Factor Loadings** |  |  |  |
| CADe Trust | 0.824 |  |  |  |
| CADx Trust | 1.165 |  |  |  |
| CADi Trust | 0.827 |  |  |  |
|  |  |  |  |  |
|  |  |  |  |  |
| **Consistency and Unidimensionality Check for Risk Perception** | | | |  |
| Internal Consistency: Omega Hierarchical | | |  |  |
| **Sample Size** | **Estimate** | **Std Error** | **95% Lower Limit** | **95% Upper Limit** |
| 165 | 0.897 | 0.018 | 0.859 | 0.930 |
|  |  |  |  |  |
| Internal Consistency: Pearson r | |  |  |  |
| **Lowest r** | **Highest r** | **0.25 quantile r** | **Median r** | **0.75 quantile r** |
| 0.629 | 0.822 | 0.691 | 0.753 | 0.787 |
|  |  |  |  |  |
| Test of Unidimensionality: Confirmatory Factor Analysis | | |  |  |
| **Factor** | **Factor Loadings** |  |  |  |
| CADe Risk | 1.259 |  |  |  |
| CADx Risk | 1.507 |  |  |  |
| CADi Risk | 1.175 |  |  |  |

**Result 3 Expanded Regression Analysis Results**

Trust is the dependent variable. Here we compare regression results with and without the interaction term. Interaction term is defined as the product of Risk Perception and Acceptance.

Interpretation of the results below:

Scenario 1: CADe

R2: Modest for both models (with and without the interaction term) yet deemed satisfactory.

p-value: Notable for all variables, irrespective of the presence of the interaction term.

Conclusion: Trust can be predicted by Risk Perception (RP), Acceptance, and the interaction term.

Scenario 2: CADx

R2: Exceeds that of scenario 1.

p-value: For the model excluding the interaction term, both RP and Acceptance are notable. However, only Acceptance stands out in the model containing the interaction term.

Conclusion: The influence of RP on Trust is ambiguous. In contrast, Acceptance evidently predicts Trust.

Scenario 3: CADi

R2: Highest of 3 scenarios.

p-value: Acceptance is notable, but RP loses its significance entirely.

Conclusion: Trust is evidently influenced by Acceptance.

From the provided scenarios, we can derive the following conclusions about the interaction between Risk Perception (RP), Trust, and Acceptance:

**Consistent Influence of Acceptance:** In all three scenarios, Acceptance consistently shows significance in predicting Trust, regardless of the presence or absence of the interaction term. This indicates that Acceptance is a robust predictor of Trust across different scenarios.

**Ambiguity of RP's Influence:** In Scenario 1, RP is significant in predicting Trust, both with and without the interaction term. However, in Scenario 2, its influence becomes unclear, especially in the presence of the interaction term. By Scenario 3, RP no longer shows any significant influence on Trust. This suggests that RP's influence on Trust is context-dependent and might be affected by other factors or variables not captured in these scenarios.

**Interaction Term's Influence:** In Scenario 1, the interaction term between RP and Acceptance is significant, implying that the effect of RP on Trust could vary depending on Acceptance levels (or vice versa). This indicates that their combined effect on Trust isn't simply additive. In Scenarios 2 and 3, the interaction term is no longer significant, suggesting that in these specific contexts, there isn't a significant combined effect of RP and Acceptance on Trust beyond their individual impacts.

**Overall Conclusion:** Acceptance has a direct and consistent influence on Trust across the board. On the other hand, RP's influence on Trust seems to be more context-dependent. The interaction between RP and Acceptance is significant in Scenario 1, suggesting potential context-specific interdependencies between these factors. However, this combined influence is not observed in Scenarios 2 and 3, emphasizing the need to consider the application of the AI when analyzing their joint impact.

| **OLS Regression Results for CADe without Interaction Term** | | | |  |  |  |
| --- | --- | --- | --- | --- | --- | --- |
| **Dep. Variable:** | Trust | **R-squared:** | 0.207 |  |  |  |
| **Model:** | OLS | **Adj. R-squared:** | 0.197 |  |  |  |
| **Method:** | Least Squares | **F-statistic:** | 21.1 |  |  |  |
| **Date:** | Tue, 24 Oct 2023 | **Prob (F-statistic):** | 7.19E-09 |  |  |  |
| **Time:** | 11:08:09 | **Log-Likelihood:** | -223.85 |  |  |  |
| **No. Observations:** | 165 | **AIC:** | 453.7 |  |  |  |
| **Df Residuals:** | 162 | **BIC:** | 463 |  |  |  |
| **Df Model:** | 2 |  |  |  |  |  |
| **Covariance Type:** | nonrobust |  |  |  |  |  |
|  |  |  |  |  |  |  |
|  | **coef** | **std err** | **t** | **P>\|t\|** | **[0.025** | **0.975]** |
| **const** | 3.8536 | 0.471 | 8.175 | 0 | 2.923 | 4.784 |
| **Acceptance** | 0.4111 | 0.071 | 5.768 | 0 | 0.27 | 0.552 |
| **Risk Perception** | -0.1091 | 0.049 | -2.226 | 0.027 | -0.206 | -0.012 |
|  |  |  |  |  |  |  |
| **Omnibus:** | 30.634 | **Durbin-Watson:** | 1.87 |  |  |  |
| **Prob(Omnibus):** | 0 | **Jarque-Bera (JB):** | 54.667 |  |  |  |
| **Skew:** | -0.9 | **Prob(JB):** | 1.35E-12 |  |  |  |
| **Kurtosis:** | 5.17 | **Cond. No.** | 45.4 |  |  |  |
|  |  |  |  |  |  |  |

|  |  |  |  |  |  |  |
| --- | --- | --- | --- | --- | --- | --- |
| **OLS Regression Results for CADe with Interaction Term** | | | |  |  |  |
| **Dep. Variable:** | Trust | **R-squared:** | 0.236 |  |  |  |
| **Model:** | OLS | **Adj. R-squared:** | 0.221 |  |  |  |
| **Method:** | Least Squares | **F-statistic:** | 16.54 |  |  |  |
| **Date:** | Tue, 24 Oct 2023 | **Prob (F-statistic):** | 2.03E-09 |  |  |  |
| **Time:** | 11:08:10 | **Log-Likelihood:** | -220.77 |  |  |  |
| **No. Observations:** | 165 | **AIC:** | 449.5 |  |  |  |
| **Df Residuals:** | 161 | **BIC:** | 462 |  |  |  |
| **Df Model:** | 3 |  |  |  |  |  |
| **Covariance Type:** | nonrobust |  |  |  |  |  |
|  |  |  |  |  |  |  |
|  | **coef** | **std err** | **t** | **P>\|t\|** | **[0.025** | **0.975]** |
| **const** | 0.6464 | 1.378 | 0.469 | 0.64 | -2.076 | 3.368 |
| **Acceptance** | 0.9415 | 0.226 | 4.169 | 0 | 0.496 | 1.387 |
| **Risk Perception** | 0.653 | 0.312 | 2.092 | 0.038 | 0.037 | 1.269 |
| **InteractionT** | -0.1266 | 0.051 | -2.471 | 0.015 | -0.228 | -0.025 |
|  |  |  |  |  |  |  |
| **Omnibus:** | 25.408 | **Durbin-Watson:** | 1.895 |  |  |  |
| **Prob(Omnibus):** | 0 | **Jarque-Bera (JB):** | 45.18 |  |  |  |
| **Skew:** | -0.754 | **Prob(JB):** | 1.55E-10 |  |  |  |
| **Kurtosis:** | 5.073 | **Cond. No.** | 493 |  |  |  |
|  |  |  |  |  |  |  |
|  |  |  |  |  |  |  |

| **OLS Regression Results for CADx without Interaction Term** | | | |  |  |  |
| --- | --- | --- | --- | --- | --- | --- |
| **Dep. Variable:** | Trust | **R-squared:** | 0.4 |  |  |  |
| **Model:** | OLS | **Adj. R-squared:** | 0.392 |  |  |  |
| **Method:** | Least Squares | **F-statistic:** | 53.92 |  |  |  |
| **Date:** | Tue, 24 Oct 2023 | **Prob (F-statistic):** | 1.12E-18 |  |  |  |
| **Time:** | 11:08:11 | **Log-Likelihood:** | -215.62 |  |  |  |
| **No. Observations:** | 165 | **AIC:** | 437.2 |  |  |  |
| **Df Residuals:** | 162 | **BIC:** | 446.6 |  |  |  |
| **Df Model:** | 2 |  |  |  |  |  |
| **Covariance Type:** | nonrobust |  |  |  |  |  |
|  |  |  |  |  |  |  |
|  | **coef** | **std err** | **t** | **P>\|t\|** | **[0.025** | **0.975]** |
| **const** | 2.7053 | 0.419 | 6.456 | 0 | 1.878 | 3.533 |
| **Acceptance** | 0.6406 | 0.065 | 9.803 | 0 | 0.512 | 0.77 |
| **Risk Perception** | -0.1254 | 0.046 | -2.706 | 0.008 | -0.217 | -0.034 |
|  |  |  |  |  |  |  |
| **Omnibus:** | 60.518 | **Durbin-Watson:** | 1.737 |  |  |  |
| **Prob(Omnibus):** | 0 | **Jarque-Bera (JB):** | 275.641 |  |  |  |
| **Skew:** | -1.285 | **Prob(JB):** | 1.40E-60 |  |  |  |
| **Kurtosis:** | 8.787 | **Cond. No.** | 42 |  |  |  |
|  |  |  |  |  |  |  |
|  |  |  |  |  |  |  |

| **OLS Regression Results for CADx with Interaction Term** | | | |  |  |  |
| --- | --- | --- | --- | --- | --- | --- |
| **Dep. Variable:** | Trust | **R-squared:** | 0.406 |  |  |  |
| **Model:** | OLS | **Adj. R-squared:** | 0.394 |  |  |  |
| **Method:** | Least Squares | **F-statistic:** | 36.61 |  |  |  |
| **Date:** | Tue, 24 Oct 2023 | **Prob (F-statistic):** | 4.31E-18 |  |  |  |
| **Time:** | 11:08:11 | **Log-Likelihood:** | -214.81 |  |  |  |
| **No. Observations:** | 165 | **AIC:** | 437.6 |  |  |  |
| **Df Residuals:** | 161 | **BIC:** | 450 |  |  |  |
| **Df Model:** | 3 |  |  |  |  |  |
| **Covariance Type:** | nonrobust |  |  |  |  |  |
|  |  |  |  |  |  |  |
|  | **coef** | **std err** | **t** | **P>\|t\|** | **[0.025** | **0.975]** |
| **const** | 3.9865 | 1.101 | 3.62 | 0 | 1.812 | 6.161 |
| **Acceptance** | 0.4255 | 0.183 | 2.324 | 0.021 | 0.064 | 0.787 |
| **Risk Perception** | -0.4371 | 0.252 | -1.734 | 0.085 | -0.935 | 0.061 |
| **InteractionT** | 0.0526 | 0.042 | 1.258 | 0.21 | -0.03 | 0.135 |
|  |  |  |  |  |  |  |
| **Omnibus:** | 62.169 | **Durbin-Watson:** | 1.748 |  |  |  |
| **Prob(Omnibus):** | 0 | **Jarque-Bera (JB):** | 292.613 |  |  |  |
| **Skew:** | -1.314 | **Prob(JB):** | 2.88E-64 |  |  |  |
| **Kurtosis:** | 8.972 | **Cond. No.** | 409 |  |  |  |
|  |  |  |  |  |  |  |
|  |  |  |  |  |  |  |

| **OLS Regression Results for CADi without Interaction Term** | | | |  |  |  |
| --- | --- | --- | --- | --- | --- | --- |
| **Dep. Variable:** | Trust | **R-squared:** | 0.52 |  |  |  |
| **Model:** | OLS | **Adj. R-squared:** | 0.514 |  |  |  |
| **Method:** | Least Squares | **F-statistic:** | 87.88 |  |  |  |
| **Date:** | Tue, 24 Oct 2023 | **Prob (F-statistic):** | 1.42E-26 |  |  |  |
| **Time:** | 11:08:12 | **Log-Likelihood:** | -219.97 |  |  |  |
| **No. Observations:** | 165 | **AIC:** | 445.9 |  |  |  |
| **Df Residuals:** | 162 | **BIC:** | 455.3 |  |  |  |
| **Df Model:** | 2 |  |  |  |  |  |
| **Covariance Type:** | nonrobust |  |  |  |  |  |
|  |  |  |  |  |  |  |
|  | **coef** | **std err** | **t** | **P>\|t\|** | **[0.025** | **0.975]** |
| **const** | 1.421 | 0.379 | 3.747 | 0 | 0.672 | 2.17 |
| **Acceptance** | 0.765 | 0.059 | 13.047 | 0 | 0.649 | 0.881 |
| **Risk Perception** | -0.061 | 0.047 | -1.304 | 0.194 | -0.153 | 0.031 |
|  |  |  |  |  |  |  |
| **Omnibus:** | 106.566 | **Durbin-Watson:** | 1.864 |  |  |  |
| **Prob(Omnibus):** | 0 | **Jarque-Bera (JB):** | 864.7 |  |  |  |
| **Skew:** | -2.281 | **Prob(JB):** | 1.71E-188 |  |  |  |
| **Kurtosis:** | 13.245 | **Cond. No.** | 36.2 |  |  |  |
|  |  |  |  |  |  |  |
|  |  |  |  |  |  |  |

| **OLS Regression Results for CADi with Interaction Term** | | | |  |  |  |
| --- | --- | --- | --- | --- | --- | --- |
| **Dep. Variable:** | Trust | **R-squared:** | 0.526 |  |  |  |
| **Model:** | OLS | **Adj. R-squared:** | 0.517 |  |  |  |
| **Method:** | Least Squares | **F-statistic:** | 59.53 |  |  |  |
| **Date:** | Tue, 24 Oct 2023 | **Prob (F-statistic):** | 6.01E-26 |  |  |  |
| **Time:** | 11:08:14 | **Log-Likelihood:** | -219.02 |  |  |  |
| **No. Observations:** | 165 | **AIC:** | 446 |  |  |  |
| **Df Residuals:** | 161 | **BIC:** | 458.5 |  |  |  |
| **Df Model:** | 3 |  |  |  |  |  |
| **Covariance Type:** | nonrobust |  |  |  |  |  |
|  |  |  |  |  |  |  |
|  | **coef** | **std err** | **t** | **P>\|t\|** | **[0.025** | **0.975]** |
| **const** | 0.3891 | 0.844 | 0.461 | 0.646 | -1.278 | 2.056 |
| **Acceptance** | 0.9407 | 0.141 | 6.661 | 0 | 0.662 | 1.22 |
| **Risk Perception** | 0.204 | 0.199 | 1.023 | 0.308 | -0.19 | 0.598 |
| **InteractionT** | -0.0458 | 0.033 | -1.367 | 0.173 | -0.112 | 0.02 |
|  |  |  |  |  |  |  |
| **Omnibus:** | 105.054 | **Durbin-Watson:** | 1.879 |  |  |  |
| **Prob(Omnibus):** | 0 | **Jarque-Bera (JB):** | 827.585 |  |  |  |
| **Skew:** | -2.251 | **Prob(JB):** | 1.96E-180 |  |  |  |
| **Kurtosis:** | 13.006 | **Cond. No.** | 296 |  |  |  |
